# Ceruloplasmin and Copper: Méndez and Araya Respond

**Published:** 2005-04

**Authors:** Marco Méndez, Magdalena Araya

**Affiliations:** University of Chile Institute of Nutrition and Food Technology, University of Chile, Santiago, Chile, E-mail: mmendez@inta.cl

Most unfortunately, Tweedale misunderstood and misinterpreted our article “‘Sex and Ceruloplasmin Modulate the Response to Copper Exposure in Healthy Individuals” (Mendéz et al. 2004). The article is about copper homeostasis and homeostatic regulation, not about toxic effects associated with copper. The dose administered was 10 mg/day and not 10 mg/kg/day, which we agree may be toxic. The dosage and the time of study used allowed us to assess homeostatic mechanisms in normal human beings; this study will contribute to the development of specific recommendations for subgroups of the population that have genetic polymorphisms that render them more susceptible to minor copper deficiency and copper excess.

Aminotranferases are the current gold standard to assess liver damage; therefore, it is correct that although no adverse effect was expected because the dose is safe, ethical considerations made it pertinent to measure their activities. These enzymatic activities are routinely measured for diagnostic purposes in individuals who manifest symptoms of illnesses. It is not known whether they reflect minor changes in hepatic function when enzymatic values are within the normal limits and there is no illness. Thus, it was most interesting to assess the potential of these enzymes to detect changes within the normal range in the studied individuals.

Certainly, our work is based on a series of concepts that include the upper safe limit (which represents the safe chronic average intake of the metal for human beings), tolerable daily intake (TDI), and several others [e.g., dietary allowances, adequate recommended oral intake (AROI), lower concentration of observed effects (LOEL)] that served to ensure that the protocol was within safe limits for human adults. Although approximately 2–2.5% of the normal population is not included in some of the concepts mentioned, this does not mean that this percentage will be damaged by the exposure but that they represent individuals with illnesses, and therefore are not included among the normal population, who require a different treatment. All these concepts, of course, are not “typically derived from industry junk science (unpublishable in independent journals)” and they do not “contain massive data gaps,” as stated by Tweedale, but they do represent the state of the art on a specific topic produced by experts appointed by the National Institutes of Health, the World Health Organization, and other respected agencies.

Our study (Mendéz et al. 2004) indeed followed the ethical considerations required to work with human subjects. The protocol was approved by our institutional review board, which is registered (IRB00001493) with the Office for Human Research Protections.

Last and most important, how to deal with potential conflicts of interest in science is a hot topic, and there is currently no final solution (Blumenthal 2003; Morin et al. 2002; Nathan and Wilson 2003; Tufts University 2003). We made full disclosure of our financial support, and it is up to the readers to judge the situation for themselves.

## References

[b1-ehp0113-a0226b] Blumenthal D (2003). Academic-industrial relationships in the life sciences. N Eng J Med.

[b2-ehp0113-a0226b] Méndez MA, Araya M, Olivares M, Pizarro F, Gonzalez M (2004). Sex and ceruloplasmin modulate the response to copper exposure in healthy individuals. Environ Health Perspect.

[b3-ehp0113-a0226b] Morin K, Rakatanska H, Riddick FA, Morse LJ, O’Bannon JM, Goldrich MS (2002). Managing conflicts of interest in the conduct of clinical trials. JAMA.

[b4-ehp0113-a0226b] Nathan DG, Wilson JD (2003). Clinical research and the NIH-A report card. N Engl J Med.

[b5-ehp0113-a0226b] Tufts University 2003. Conflict of Interest Policy. Available: http://techtransfer.tufts.edu/tufts/pol_guide/conflict.html [accessed 1 March 2005].

